# Adrenal Tumors in Children and Adolescents in Sweden: A Register-Based Study Covering 15 Years

**DOI:** 10.1210/jendso/bvaf058

**Published:** 2025-04-02

**Authors:** Eleni Terezaki, Jan Calissendorff, Buster Mannheimer, Jonatan D Lindh, Henrik Falhammar

**Affiliations:** Department of Endocrinology, Karolinska University Hospital, SE-17176 Stockholm, Sweden; Centre for Inherited Metabolic Diseases, Karolinska University Hospital, SE-171 76 Stockholm, Sweden; Department of Endocrinology, Karolinska University Hospital, SE-17176 Stockholm, Sweden; Department of Molecular Medicine and Surgery, Karolinska Institutet, SE-171 76 Stockholm, Sweden; Department of Clinical Science and Education Södersjukhuset, Karolinska Institutet, SE-171 77 Stockholm, Sweden; Department Laboratory Medicine, Division of Clinical Pharmacology, Karolinska Institutet, SE-141 52 Stockholm, Sweden; Department of Endocrinology, Karolinska University Hospital, SE-17176 Stockholm, Sweden; Department of Molecular Medicine and Surgery, Karolinska Institutet, SE-171 76 Stockholm, Sweden

**Keywords:** adrenocortical cancer, neuroblastoma, pheochromocytomas, adrenal metastases, functional adrenal tumor, mortality

## Abstract

**Context:**

Adrenal tumors (ATs) are highly uncommon in children and adolescents, and more information on these tumors is needed.

**Objective:**

The aim of this study was to describe the tumor incidence, patient and tumor characteristics, treatment, and mortality in pediatric patients with ATs.

**Methods:**

This is a Swedish nationwide, register-based, retrospective study. All patients up to 21 years old diagnosed between 2005 and 2019 with an AT were identified through national registers and then manually reviewed. Age-, sex-, and municipality-matched controls in a ratio 4:1 were selected from the total population register.

**Results:**

In total, 230 patients were included (and 920 controls), with an annual incidence of 6.20 new ATs per million for individuals up to 21 years old. The median age was 6.0 years (interquartile range, 1.0-17.70), with 120 (52.2%) being boys. Regarding tumor biology, 132 (57.4%) were malignant, 77 (33.5%) benign, and 21 (9.1%) were undetermined. There were at least 39 (16.9%) hormonally active ATs recognized as either pheochromocytomas, adrenocortical carcinomas, or benign functional adenomas. Patients with malignant tumors were younger than patients with benign tumors (mean age 2 vs 18, *P* < .001). Among patients with malignant ATs, the mortality reached 33.3% during a follow-up period of up to 15 years. Patients who were younger and received less aggressive treatments had better overall survival. Mortality was increased in all patients with malignant ATs compared to controls (*P* < .0001). Mortality was similar between patients with benign ATs and controls (*P* > .05).

**Conclusion:**

Although rare, most identified tumors were malignant and associated with high mortality.

In the pediatric and adolescent population, adrenal tumors (ATs) constitute a rare and heterogeneous group of diseases. They originate either from the medulla, as various neuroblastic tumors or pheochromocytomas, or from the adrenal cortex as adrenocortical carcinomas (ACCs) or adenomas. Unlike the ATs in adults, which are found in high numbers and are mostly benign and hormonally inactive [1, 2], the pediatric ATs are often malignant [3] or commonly present with a hormonal activity [4, 5]. These differences can be attributed to the embryonic origin of pediatric ATs, like neuroblastomas derived from neural crest tissue or some ACCs from the androgen-producing fetal zone [6]. There is also a high prevalence of genetic variants in pediatric malignances [5, 7, 8]. Moreover, pediatric ATs display a variety of clinical presentations, from tumors with spontaneous regression to rapidly metastasizing disease with high mortality. Although there are some registries on pediatric malignant ATs [4, 9], and in specific populations with founder gene variants [10] or analyses from tertiary referral centers [11, 12], there is no systematic analysis of all pediatric ATs, patient characteristics, and outcomes on a nationwide scale.

The aim of this study was to describe the distribution and incidence of ATs in the entire Swedish children and adolescent population. Further, we wanted to gather information using multiple national registries about the incidence, characteristics of the patients, and describe the treatments along with data pertinent to the endocrine or the malignant nature of ATs. Lastly, the aim was also to analyze the mortality in this population and determine some of the characteristics that relate to survival.

## Methods

**Figure 1.**
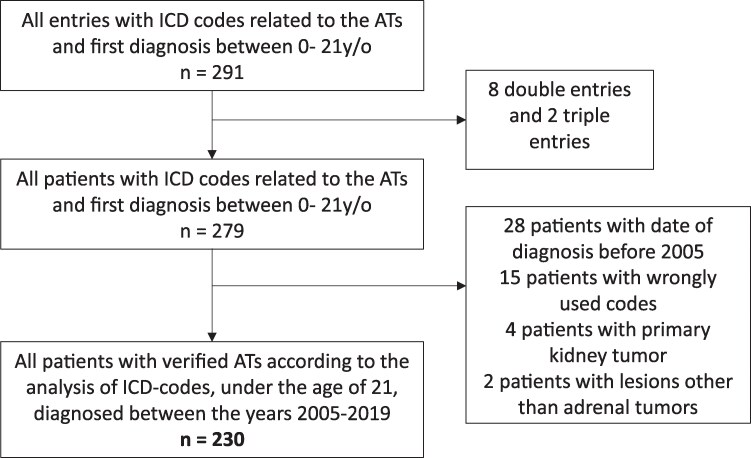
Flow chart of the study. The ratio of cases to controls was 1:4.

This was a Swedish nationwide retrospective study based on the analysis of multiple registries. The combination of data from multiple sources was possible thanks to the use of the 12-digit Swedish personal identity number that was used as a connection link between registries. The cohort of patients was initially selected using the National Patient Register, which includes International Classification of Diseases 10 (ICD10) codes from all inpatient and outpatient specialist contacts since the year 1997. Patients with ICD10 codes that corresponded to ATs, with a first ever AT diagnosis registered between 1 January 2005 and 31 December 2019, and age between 0 and 21 at the time of first registration were included in the cohort (Fig. 1). The age limit was adapted according to the design of similar studies and the US Food and Drug Administration (FDA) [3]. Using the FDA recommendations, children were categorized between the ages of 0-11 years old and adolescents in the age group 12-21 years old. The ICD10 codes used were C740 (Malignant neoplasm of cortex of adrenal gland), C741 (Malignant neoplasm of medulla of adrenal gland), C749 (Malignant neoplasm of unspecified part of adrenal gland), C797 (Secondary malignant neoplasm of adrenal gland), D350 (Benign neoplasm of adrenal gland), and D441 (Neoplasm of uncertain behavior of adrenal gland). Additional information about the patients was retrieved from 3 other registries. The causes and dates of death were found using the Cause of Death Register between years 2005 and 2020. The National Prescribed Drug Register provided information from 1 July 2005 to 31 December 2020 about all the medications that were ever dispensed and date of dispensing using Anatomical Therapeutic Chemical (ATC) codes, although over the counter medications or medications during hospital stay are not included. Finally, using the National Cancer Register (1958-2019) we determined the cell type and biological behavior of tumors, when possible. Due to lack of access to the actual medical records, all the registries were reviewed manually and in chronological fashion for each case, in order to try to reconstruct the medical history of each patient, from first warning symptom leading to a specialist visit and the types of treatment and consequent side effects, until the end of follow-up. The appropriate ICD10 codes, the pattern of follow-up visits, the types of treatments and the consequent survival of patients were decisive for the determination of the tumor's biology, with each case being reviewed by one resident of endocrinology and 1 or 2 senior specialists in endocrinology. If no conclusion about the tumors' biology could be derived beyond reasonable doubt the cases were classified as “uncertain status.” Pheochromocytomas with less than 10 years of follow-up were classified as uncertain status since many metastatic pheochromocytomas are diagnosed years after the initial diagnosis [13]. Moreover, from the open data from Statistic Sweden (available at: https://www.scb.se/en/) the number of all people 0-21 years of age residing in Sweden over the 15-year period was calculated, covering 37 091 551 person-years. For the same period, the children (0-11 years old) group's number was 19 851 939 person-years and the adolescents (12-21 years old) group's number was 17 239 612. This separation into children and adolescents was done to clarify the influence of physiological puberty on tumor behavior. Using this information, the incidence of ATs could be calculated. For each case with a new AT, 4 controls without ATs matched for sex, age, and area of residence were selected using the Total Population Registry, that is, controls could have health issues since they were from the general population but could not be diagnosed with ATs. Portions of this cohort of ATs have been used in previous studies [14-18].

The study was approved by the Swedish Ethical Review Authority. All patient information was de-identified before delivery, and due to the study’s retrospective register-based nature, all inform consents were waived.

## Statistical Analysis

Incidence was calculated using the newly diagnosed ATs dividing by the total number of people 0-21 years of age and the 2 age subgroups residing in Sweden during the study period. Descriptive statistics included numbers (percentages), range (min-max), and mean (SD) or medians (interquartile range [IQR]) as appropriate. Between the different cases, continuous variables were first analyzed using D'Agostino and Pearson tests for the determination of normality and thereafter *P* values were calculated with unpaired *t* test for normally distributed data or with Mann–Whitney *U* test for data without normal distribution. Binary variables were analyzed using the Fisher exact test. For the presentation of survival, Kaplan-Meier curves were used. Survival analysis was first made by Cox regression but if no case of death in the controls were detected, log-rank test was used. A *P* value less than 0.05 was considered statistically significant. Statistical analysis was conducted using GraphPad Prism 8 and R, version 4.2.2 (R Project for Statistical Computing).

## Results

### Cohort Description

During the 15-year study period, 291 cases with new ATs were diagnosed in individuals aged up to 21 years old. After manual examination of every case in the different registers, as shown in the flow-chart (Fig. 1), 230 patients were confirmed as true new ATs, with an estimated annual incidence of ATs of 6.20 per million, for this age group. The median age was 6.0 years (IQR 1.0-17.0), and 120 (52.2%) of the patients were boys. Regarding tumor biology, 132 (57.4%) of the patients had malignant ATs, 77 (33.5%) had benign ATs, and 21 (9.1%) had ATs with uncertain status. The patients with tumors categorized as of uncertain status comprised those with tumors that were either impossible to determine as malignant or benign according to ICD10 codes (n = 8), tumors treated as benign while having a malignant pathology (ganglioneuroblastoma, n = 1), or were patients with pheochromocytoma (n = 12) with a follow-up of less than 10 years. There was no difference in sex distribution between patients with malignant and benign ATs. Patients with malignant ATs were younger compared to patients with benign ATs (2 years [IQR 1.0-6.0] vs 18.0 years [IQR 9.0-20.5], *P* < .001).

In descending order, the following AT groups were found: 86 (37.4%) patients with neuroblastomas, 62 (27.0%) with benign nonfunctional ATs, 20 (8.7%) with pheochromocytomas, 17 (7.4%) with ACCs, 11 (4.8%) with ganglioneuroblastomas, and 7 (3.0%) with benign functional ATs (adrenal Cushing syndrome, n = 4 [1.7%]; androgen-producing adenomas, n = 2 [0.9%]; and aldosterone producing tumor, n = 1 [0.4%]). Furthermore, 5 (2.2%) patients had metastases to the adrenals and 2 (0.9%) had ganglioneuromas. Of the remainder, 12 (5.2%) patients had unclassified malignant ATs and 8 (3.5%) patients had ATs of uncertain behavior. In Table 1, the characteristics of each AT group can be seen.

**Table 1.** Characteristics of different groups of children and adolescents with adrenal tumors and types of treatment

	Incidence per 1 000 000 0-21y/o per year	Number of patients	Number of boys	Median (IQR) age of diagnosis in years	Children (0-11 y/o) Number (incidence per 1 000 000 per year)	Adolescents (12-21 y/o) Number (incidence per 1 000 000 per year)	ADX total	Chemotherapy total	Chemotherapy and ADX	No treatment
NB	2.32	86 (37.4%)	47 (54.7%)	1.5 (1-3)	84 (4.23)	2 (0.12)	34 (39.5%)	81 (94.3%)	31 (36.0%)	2 (2.3%)
Benign NFATs	1.67	62 (27.0%)	36 (58.1%)	18.5 (8.75-21)	18 (0.91)	44 (2.55)	9 (14.5%)	0	0	53 (85.5%)
PHEO	0.54	20 (8.7%)	10 (50.0%)	17 (14.25-19)	3 (0.15)	17 (0.99)	20 (100%)	2 (10%)	2 (10%)	0
ACC	0.46	17 (7.4%)	7 (41.2%)	6 (2-14.5)	11 (0.55)	6 (0.35)	16 (94.1%)	10 (58.8%)	9 (52.9%)	0
GNB	0.30	11 (4.8%)	4 (36.4%)	4 (3-7)	10 (0.50)	1 (0.06)	5 (45.5%)	7 (63.7%)	4 (36.4%)	3 (27.3%)
Benign functional adenoma	0.19	7 (3.0%)	2 (28.8%)	13 (8-21)	3 (0.15)	4 (0.23)	7 (100%)	0	0	0
Adrenal metastases	0.13	5 (2.2%)	1 (20.0%)	21 (18-21)	0	5 (0.29)	0	5 (100%)	0	0
GN	0.05	2 (0.9%)	2 (100%)	9 (2-16)	1 (0.05)	1 (0.06)	2 (100%)	1 (50.0%)	1 (50.0%)	0
Unspecified malignancy	0.32	12 (5.2%)	7 (58.3%)	2 (1-6.75)	11 (0.55)	1 (0.06)	4 (33.3%)	10 (83.3%)	3 (25.0%)	1 (8.3%)
Unknown dignity	0.22	8 (3.5%)	4 (50%)	13 (3-18.75)	4 (0.20)	4 (0.23)	0	0	0	8 (100%)
Average/Total	6.20	230 (100%)	120 (52.2%)	6 (1-17.75)	145 (7.30)	85 (4.93)	97 (42.2%)	116 (50.4%)	50 (21.7%)	67 (29.1%)
Total benign	2.08	77 (33.5%)	42 (52.2%)	18 (9-20.5)	23 (1.16)	54 (3.13)	23 (29.9%)	0	0	54 (70.1%)
Total malignant	3.56	132 (57.4%)	69 (52.3%)	2 (1-6)	115 (5.79)	17 (0.99)	62 (47.0%)	116 (87.9%)	50 (37.9%)	4 (3%)
Total uncertain	0.57	21 (9.1%)	9 (42.9%)	17 (8.5-19)	7 (0.35)	14 (0.81)	12 (57.1%)	0	0	9 (42.9%)
*P* value benign vs malignant	—	—	0.7754	<0.0001	—	—	0.0193	<0.0001	<0.0001	<0.0001

Abbreviations: ACC, adrenocortical carcinoma; ADX, adrenalectomy; GN, ganglioneuroma; GNB, ganglioneuroblastoma; IQR, interquartile range; NB, neuroblastoma; NFAT, nonfunctional adrenal tumor; PHEO, pheochromocytoma.

### Most Common Adrenal Tumors

The most frequent AT diagnosis was neuroblastoma (n = 86), with an estimated annual incidence of 2.32 patients per million. No differences were found in sex distribution of patients with neuroblastoma and age at diagnosis (*P* = 0.189). Patients with neuroblastomas were younger at diagnosis than patients with other malignant ATs (1.5 [IQR 1.0-3.0] vs 6.0 [IQR 2.0-15.0] years, *P* < .0001). It was more common that neuroblastomas progressed into recurrent or metastatic disease compared with the other malignant ATs (77.9% vs 56.5%, *P* = .016). The overwhelming majority of patients with neuroblastoma received chemotherapy, compared to patients with other AT malignancies (94.3% vs 82.6%, *P* = .062), while isotretinoin was added in the regiment more often in the treatment of neuroblastomas (51.2% vs 13%, *P* < .001). Patients with other AT malignancies were more likely to receive adrenalectomy (60.9% vs 39.5%, *P* = .028).

The second most frequent category was the benign nonfunctional ATs (n = 62), with an annual incidence of 1.67 per million among those 0-21 years of age. There were no differences in sex distribution (*P* = .474) or age at diagnosis with median age in benign ATs being similar between sexes (girls 18.5 [IQR 11.25-21] vs boys 18.5 [IQR 6.5-21], *P* = .633). Adrenalectomy was performed in 14.5% of patients and the rest of benign nonfunctional ATs were managed conservatively.

### Hormone-Producing Adrenal Tumors

In the cohort, 20 patients (8.7% of the entire cohort) were recognized as having pheochromocytoma, of whom 2 had metastatic pheochromocytoma, 6 had a benign variant (no recurrence during at least 10 years of follow-up), and 12 patients with tumors of uncertain behavior due to a follow-up period of less than 10 years until 2019. Of all patients with pheochromocytomas, 16 patients had hypertension at presentation, and of these, the majority (n = 12, 75%) seemed to be normotensive after adrenalectomy. All patients with pheochromocytomas were treated with adrenalectomy, 2 required bilateral, and the most common postoperative complication was adrenal insufficiency (n = 8, 40%). No data were recorded on if cortical-sparing adrenalectomy was performed. At least half of the patients had a known genetic syndrome before the diagnosis of pheochromocytoma, and until the end of our observation period at least 13 (65%) were diagnosed with von Hippel-Lindau syndrome, neurofibromatosis, or multiple endocrine neoplasia type 2 (MEN2). Nine patients also presented with additional tumors, the most common being thyroid cancers (n = 5); of these, 4 were confirmed to be medullary thyroid cancer (all diagnosed with MEN2). Excluding the patients whose pheochromocytomas were discovered during planned follow-up because of a known genetic syndrome, the rest had a mean delay of 141.4 days (range, 36-600 days) between the first visits to a specialist unit regarding warning symptoms until the adrenalectomy.

ACC was the fourth most common diagnosis (n = 17), accounting for an annual incidence of 0.46 per million. At least 70% could be confirmed as having hormonally active ACC with three-fourths producing cortisol and one-fourth androgens. Among 9 patients with cortisol-producing ACC, 6 presented with Cushing syndrome. The mean delay between first warning symptoms recorded by a specialist unit or emergency department until the tumor diagnosis was 13 days (range 0-40). None of the patients with ACC had a concomitant diagnosis of Li-Fraumeni or Beckwith-Wiedemann syndrome. All but one patient were treated with adrenalectomy, while chemotherapy was administered in 10 (58.8%) and 9 (52.9%) of them received complementary mitotane. Three-fourths of the patients (n = 13) developed adrenocortical insufficiency during the course of their treatment with 9 of them diagnosed directly postoperatively. Moreover, 4 patients receiving mitotane developed hypothyroidism.

From the 7 patients with benign functional adenomas, it could be determined that 4 (1.7%) had adrenal Cushing syndrome, 2 (0.9%) had androgen-producing adenomas, and 1 (0.4%) had primary aldosteronism. All patients with functional adenomas were treated with unilateral adrenalectomy that led to resolution of symptoms. The median time between the first contacts with a specialist unit regarding warning symptoms until surgery was 116.8 days (range, 12-390 days). All patients with adrenal Cushing syndrome developed temporary adrenal insufficiency postoperatively with the median time between first and last refill of hydrocortisone prescription being 320.5 days (range, 134 to 569 days).

### Mortality

**Figure 2.**
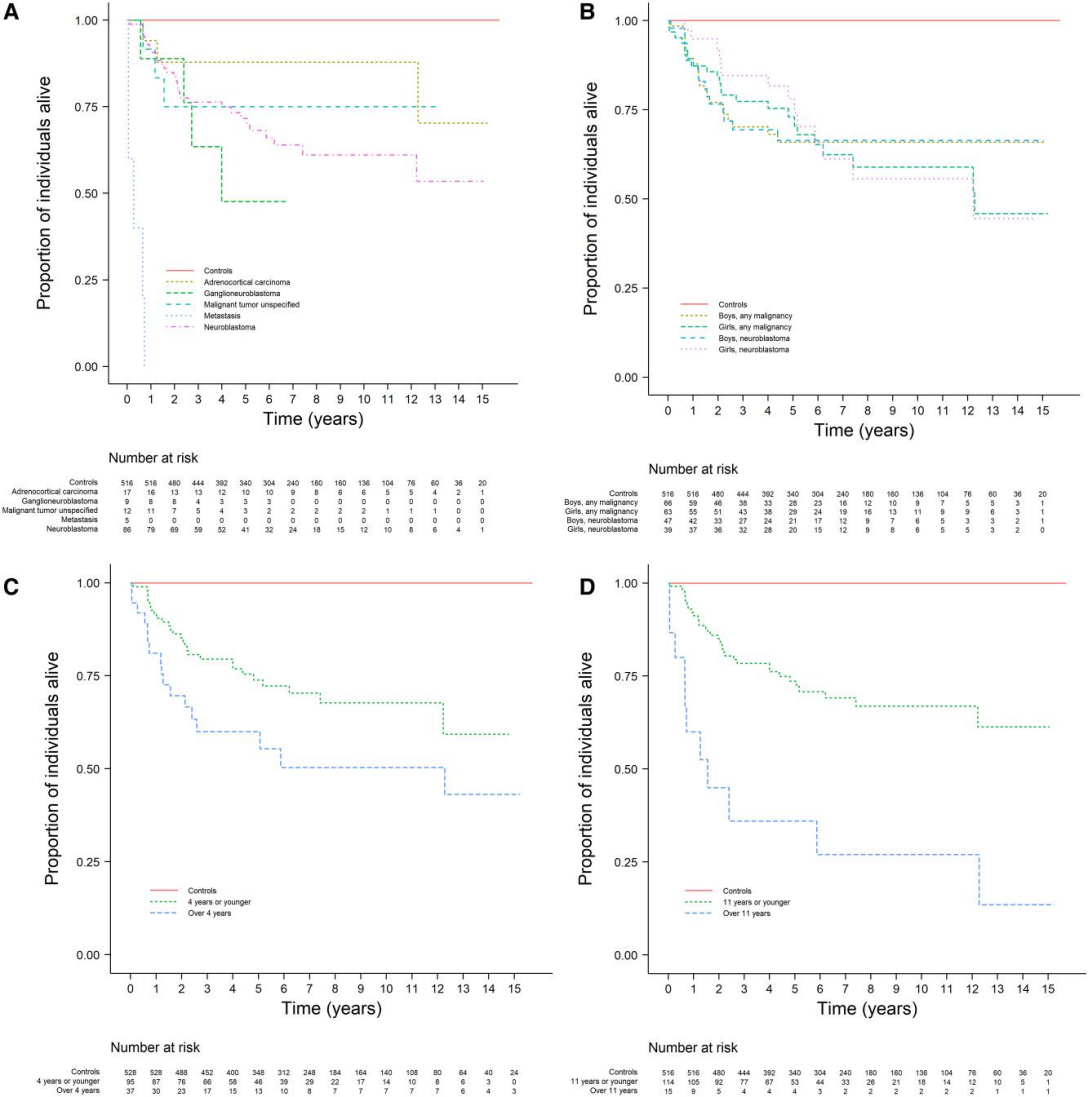
Kaplan-Meier curves of overall survival for all patients with malignant adrenal tumors compared to age-, sex-, and place-of-residence-matched controls; stratified by type of tumor (A), sex (B), and age at diagnosis (C, D). Metastasis is adrenal metastases from nonadrenal cancers. All *P* < .0001 compared to matched controls.

Of the total 230 patients of our cohort, the mortality reached 19.6%, as compared to 0% in 920 controls (*P* < .001) (Table 2). No deaths were found among patients with benign ATs and there was only 1 death among patients with uncertain ATs (a patient with pheochromocytoma), attributed to a concomitant CNS malignancy. Finally, the mortality between patients with malignant ATs reached 33.3% (44 deaths), which was higher than in controls (*P* < .001) (Fig. 2A).

**Table 2.** Mortality during follow-up in different groups of children adolescents with adrenal tumors

	Mortality	Number of deceased boys	Median (min-max) days of survival	*P* value survival days boys vs girls	*P* value age of diagnosis between alive vs deceased
NB	29 (33.7%)	15 (51.7%)	750 (35-4459)	.0025	.0024
Benign NFATs	0	—	—	—	—
PHEO	1 (5%)	0 (0%)	919	—	—
ACC	3 (17.6%)*^a^*	1 (33.3%)	462 (242-4481)	—	.0106
GNB	4 (36.4%)	2 (50%)	933 (203-1457)	ns	ns
Functional adenoma	0	—	—	—	—
Adrenal metastases	5 (100%)*^a^*	1 (20%)	98 (17-263)	—	—
GN	0	—	—	—	—
Unspecified malignancy	3 (25%)	2 (66.7%)	337 (245-567)	—	ns
Unknown dignity	0	—	—	—	—
Average	45 (19.6%)	21 (46.7%)	567 (17-4481)	ns	ns
Total benign	0	—	—	—	—
Total malignant	44 (33.3%)	21 (47.7%)	567 (17-4481)	ns	.0009
*P* value benign vs malignant	<.0001	—	—	—	—

Abbreviations: ACC, adrenocortical carcinoma; ADX, adrenalectomy; GN, ganglioneuroma; GNB, ganglioneuroblastoma; NB, neuroblastoma; NFAT, nonfunctional adrenal tumor; PHEO, pheochromocytoma.

^a^All deaths occurred in adolescents and 50% of adolescents with ACC and 100% of adrenal metastases died during follow-up.

Of the 44 patients deceased due to malignant ATs, 21 (47.7%) were boys and there was no difference in mortality between boys vs girls (*P* = .467) or age of diagnosis dependent on patients' sex (*P* = .109). Deceased girls had a longer median survival with the difference driven exclusively by girls with neuroblastoma (*P* = .003) (Fig. 2B). Between patients with malignant ATs, there was a difference in age of diagnosis and mortality, with the group of surviving patients being younger than the group of deceased (mean age 3.37 vs 6.54 years, *P* = .001) (Fig. 2C).

The survival rate among patients with primary malignant ATs (not including adrenal metastases) differed depending on the types of treatment. Mortality among patients who only needed adrenalectomy was 0% (0/12), for those treated only with chemotherapy it was 21% (8/38), chemotherapy and adrenalectomy 39% (9/23), chemotherapy and isotretinoin 39% (9/23), adrenalectomy, chemotherapy, and isotretinoin 44% (12/27) and no known treatment 25% (1/4). In our cohort, all patients with metastasis to the adrenals died shortly after the metastasis was detected, regardless of primary tumor's treatment.

## Children vs Adolescents

The cohort included 145 children with ATs and 85 adolescents with ATs. The estimated annual incidences in these 2 groups were 7.30 and 4.93 per million per year, respectively. The number of individuals and incidences of the different AT types can be seen in Table 1. When comparing mortality in children and adolescents with malignant ATs, the mortality was higher among adolescents compared to children (*P* = .0053) (Fig. 2D). This was driven by the fact that all adolescents with adrenal metastases and half with ACC succumbed.

## Discussion

This is the first nationwide study of all malignant and benign ATs in pediatric and adolescent population and included 230 patients. Due to the register-based method employed and the long observation period, it was possible to collect data on both the malignant and the lesser-studied benign ATs despite their rarity in children and adolescents. Thus, we were able to estimate the annual AT incidence at 6.20 per million in Sweden in children and adolescents (7.30 in children and 4.93 in adolescents). Unlike the adult population, where the overwhelming majority of detected ATs are benign nonfunctional [2, 19-21], in the pediatric population it was found that more than half were malignant ATs, mostly detected early in life, as well as many hormonally active ATs. Of the total cohort, 42.2% of patients were treated with adrenalectomy and of the patients with malignant ATs, adrenalectomy was performed in 47%, with 87.9% receiving chemotherapy. The mortality reached 19.6%, which was much higher compared to controls and was exclusively attributed to malignant ATs. In malignant ATs, the type of treatment, which can be used as a surrogate for the stage of the malignancy, correlated with survival.

The majority of previous studies on pediatric ATs were performed in single centers [11, 12] or focused only on patients who received adrenalectomy [3, 22-24]. Thus, the incidence of pediatric ATs has not been estimated before in the general population. Most of these cohorts reported that malignant ATs were more prevalent than benign. In studies that included histopathology it was evident that neuroblastoma was the most prevalent diagnosis. Only in Zhang et al, adrenal hematomas were found to be the most common adrenal mass with the majority detected before 1 month of age and after trauma [11]. The reason for this discrepancy between the study by Zhang et al and most other cohorts is unclear but may include more frequent abdominal ultrasounds in neonates, more childhood traumas, and more infections in the former study. Moreover, in our study, the ICD10 diagnosis for adrenal hemorrhage in neonates was not included in the studied diagnoses. As with our study, in the majority of studies, malignant ATs were more prevalent in the younger patients [3, 11, 12, 23] and there was a tendency toward boys being more affected [11, 12, 22, 24]. The tendency was also observed in large cohorts of malignant ATs [9, 25].

Neuroblastomas account for 8% to 10% of all childhood malignances and emerge from neural crest-derived cells that underwent defective differentiation [8]. The most common site is the adrenal medulla, at around 63%, while neuroblastomas can also be found in the sympathetic ganglia of the neck, chest, or abdomen (including pelvis) [25]. The annual incidence of adrenal neuroblastoma in our study was estimated at 2.32 per million in individuals up to 21 years old and at 4.23 per million in children from 0 to 11 years old, although the number could be higher if some of the 20 patients with “unclassified malignant ATs” or “ATs of uncertain behavior” were neuroblastomas. According to a SEER registry analysis [25], the incidence of neuroblastoma and ganglioneuroblastoma, of all sites, in children (defined as ≤14 years) was higher at 5.0 to 8.5 per million person-years, while for adolescents/adults (>14 years) it was only 0.1 to 0.5, the latter similar to the incident of 0.12 found in our adolescents. The lower number in our data may be explained by the inclusion of adrenal neuroblastomas only together with the wider age range. In our cohort, 85 out of 86 patients with adrenal neuroblastomas and all 11 patients with ganglioneuroblastoma were ≤14 years, with the estimated incidence considering only the ≤14-year-olds reaching 3.4 per million-years (or 3.87 with both neuroblastomas and ganglioneuroblastomas), which is comparable to the incidence of abdominal neuroblastomas (63%) in the SEER study. In another large cohort of multiple malignant ATs [9] neuroblastomas were also found more frequently among younger patients and having a higher percentage of metastatic disease in all age groups compared to ganglioneuroblastoma, ACC, and other malignant ATs. Unlike Lv et al [9], we found that ganglioneuroblastoma and neuroblastoma and not ACC had the highest mortality among malignant ATs, which potentially could be explained by less biases in our study. Moreover, around half of all cases of pediatric ACC are reported from South Brazil due to a founder effect of germline *TP53-R337H* variant [10, 26], that is, a publication bias. The annual incidence of pediatric ACC has a wide range, from 0.18 per million in European cohorts [27] to up to 12- to 18-times higher in Brazil [28]. Factors such as age ≤4 years, localized disease, complete surgical resection [29-31], and even lack of *TP53* variants [32] have been associated with exceptional survival. It is unclear why our patients with ACC in the younger group displayed such good survival, but possible explanations could include the relatively small number of patients, the genetic makeup and variants of the tumors, that three-fourths of adrenalectomies were performed at tertiary university hospitals, and the possible misclassification of benign adrenocortical adenomas as ACC [33, 34]. However, half of our adolescents with ACC died during follow-up, thus in this age group the prognosis was poor. None of the patients with ACC in the present study had a concomitant diagnosis of Li-Fraumeni or Beckwith-Wiedemann syndromes, which have been associated with pediatric ACC [35]. Moreover, no other malignancy was diagnosed under our observation period, contrasting our results for pheochromocytoma. In accordance with the literature [4], at least 70% of children in the current study had hormonally active ACC, though cortisol production was more prevalent than androgen production. Since we did not have access to the patients' medical files or the laboratory and pathology reports, it is possible that a larger percent of patients had indeed androgen excess but lacked the appropriate ICD10 codes.

Both incidence and median age of diagnosis are within the well-characterized range for pheochromocytoma [36]. As previously mentioned, young patients with pheochromocytomas have a higher percentage of hereditary disease than adults [5, 37]. In our study, at least 65% could be confirmed to have a familial syndrome, which is probably an underestimation, since there is no ICD10 code for succinate dehydrogenase complex or many other genetic variants that result in pheochromocytoma syndromes. Both bilateral and metastatic pheochromocytomas were recognized in our cohort at 10%, which is lower than what was described in the large cohort of Pamporaki et al [37].

Although it was not our primary endpoint, we were able to identify children and adolescents with AT-related Cushing syndrome, amounting to 10 patients, or 6 with ACC and 4 with benign ATs. The incidence of endogenous adrenal Cushing syndrome in this age group is by no means complete, since many syndromes related to adrenal hyperplasia were not part of our inclusion criteria [38].

The literature is also scarce on reports about benign nonfunctional ATs in the general population in this age group. Unfortunately, none of our patients had an accompanying histological diagnosis to differentiate between adenomas, cysts, myelolipomas, and other histologically benign AT, since only a small fraction of patients with benign disease underwent adrenalectomy. Benign ATs are exceedingly rare in children, with the incidence starting to rise in late adolescence, as seen in the results of the extended pediatric adrenalectomy cohort by Uttinger et al [3]. Larger autopsy or radiological series agree that the prevalence of adrenal incidentalomas increase with age [19]. Of course, the health status of young patients who were included in those series can be assumed different from the general population.

Our results on patients with malignant ATs are comparable to other larger registry studies that have been published [9, 25]. Moreover, factors that contribute to higher survival, like younger age, localized disease, and complete surgical resection are well established [9, 25]. In our study, we lacked access to complete TNM staging or surgical records, but we could use the type of treatment and use of advanced chemotherapy schemes with isotretinoin as a surrogate of disease burden. Indeed, patients who only received surgery had an excellent prognosis, while the use of isotretinoin [39] in high-risk malignances was associated with higher mortality. Surprisingly, patients with adrenal metastases in this age group had no survival after the first year, though none of the patients could receive adrenalectomy due to the apparent high burden of disease. It should be noted that all patients with adrenal metastases were adolescents. Moreover, the adolescents had an AT profile more similar to adults and it can be speculated that puberty changed the tumor behavior to a more adult pattern. Mortality in adolescents with ATs was increased compared to children and was driven by that fact that all adolescents with adrenal metastases and half with ACC succumbed.

Some of the major limitations of the study could be that imaging modalities like computed tomography are rarely used among children, leading to a potentially substantial underestimation of small, benign, or slowly progressing ATs in this age group. Moreover, many details regarding our patients could be missing due to the use of register-based information with, for example, ICD10 and ATC codes instead of medical files. If biochemical, radiological, and detailed pathologic and genetic reports had been available it could help to determine the type and biology of tumors with greater accuracy. Thus, the functioning and nonfunctioning status of the AT should be viewed with some caution. Moreover, the presence of a genetic syndrome could also have been missed since some genetic syndromes may have been difficult to detect using ICD10 codes. Some information like TNM staging was also incomplete from the registries and was thus excluded from the analysis. Moreover, codes for radiotherapy were absent although it could be a possible therapeutic option for specific subgroups of ATs [40]. There is a possibility that some patients received treatment outside of Sweden or emigrated after diagnosis, thus compromising the accuracy of our follow-up data, but we deem this rare. Finally, we cannot account for the registration of error codes or omission of right codes, although through manual evaluation of the pathology classification given in the cancer register and the analysis of ICD10 and ATC codes in chronological order we tried to minimize the errors.

In conclusion, this is the first nationwide study to report on the incidence of all diagnosed ATs in children and adolescents. Unlike the adult population, ATs were rare and in the majority of cases malignant, particularly among the youngest, with a high mortality linked to the age of diagnosis and disease stage. Moreover, there were several malignant and benign hormone-producing ATs, with unique characteristics such as high prevalence of syndromes and concomitant tumors. Larger studies, possibly through international collaborations, are needed to investigate this rare entity in more detail.

## Grants

This project was supported by grants from the Magnus Bergvall Foundation, Karolinska Institutet, and the Stockholm County Council.

## Disclosures

The authors have nothing to disclose.

## Data Availability

Restrictions apply to the availability of data generated or analyzed during this study to preserve patient confidentiality or because they were used under license. The corresponding author will on request detail the restrictions and any conditions under which access to some data may be provided.

## Abbreviations

ACC: adrenocortical carcinoma
AT: adrenal tumor
IQR: interquartile range
